# A Rare but Life-Threatening Case of Fournier’s Gangrene Caused by Sodium-Glucose Cotransporter-2 (SGLT2) Inhibitor, Empagliflozin

**DOI:** 10.7759/cureus.29264

**Published:** 2022-09-17

**Authors:** Tahmina Jahir, Sadaf Hossain, Mobasera Bagum, Ahmed Saidi, Ruby Risal, Marie Schmidt

**Affiliations:** 1 Pulmonary Medicine, Interfaith Medical Center, Brooklyn, USA; 2 Psychiatry, Jamaica Hospital Medical Center, Queens, USA; 3 Internal Medicine, Interfaith Medical Center, Brooklyn, USA

**Keywords:** sodium-glucose cotransporter-2 (sglt-2) inhibitors, necrotizing fasciitis, fungal genital infection, uncontrolled diabetes, fournier’s gangrene

## Abstract

Fournier's gangrene (FG) is a rare but severe infection in the soft tissue, leading to necrosis in the perineum, perianal and genitourinary area. This infection can spread rapidly in the body and lead to multi-organ failure, septic shock, and death. This life-threatening infection is usually caused by polymicrobial agents like Group A - Beta Hemolytic Streptococcus- Streptococcus pyogenes, Staphylococcus aureus, Escherichia coli, Klebsiella pneumonia, Proteus, and anaerobes like Bacteroides and Clostridium perfringes. Risk factors related to the development of FG are obesity, uncontrolled diabetes, lack of education, poor personal hygiene, especially in the genital region, history of fungal infection, recurrent urinary tract infection, smoking, immunosuppression, and medication. In 2018, a safety warning was issued by The U.S. Food and Drug Administration (FDA) on sodium-glucose cotransporter-2 (SGLT2) inhibitors, causing a rare but serious adverse outcome of FG in patients with type 2 diabetes mellitus. It is established that the increased urinary glucose concentration caused by SGLT-2 inhibitors creates a suitable environment for the growth of the infection in the urinary and genital area, leading to the development of FG. Here we present a case of life-threatening FG in an obese female with a past medical history of type 2 diabetes mellitus with recurrent history of genital yeast infection four months after starting an SGLT2 inhibitor, empagliflozin. This study aims to understand the relationship between the FG and SGLT-2 inhibitor, overall the benefits of SGLT2 inhibitors outweighs the risk manyfold, therefore, raising awareness among clinician to be vigilant, keep a high index of suspicion and focus on the safe use of SGLT2 inhibitors, especially before and after prescribing SGLT-2 inhibitor with a close follow-up to prevent its serious and life-threatening emergency like Fournier's gangrene and necrotizing fasciitis.

## Introduction

Necrotizing fasciitis (NF) is a rare but highly progressive infection that can penetrate deeper soft tissue structures and cause the destruction of underlying subcutaneous fat and muscles. NF is a fatal and fast-spreading infection with a high mortality rate even with optimal therapy [1-2]. Therefore, it is crucial to understand the risk factors that contribute to NF. When it involves the perineum, perianal and genitourinary area, it is referred to as Fournier gangrene (FG). It is vital to act emergently by surgery and intervenous broad-spectrum antibiotics to prevent sepsis, septic shock, and toxic shock-like syndrome, which may lead to multi-organ failure and death [3]. Empagliflozin belongs to the SGLT2 inhibitors family, which is approved for treating type 2 diabetes mellitus (T2DM) that cannot be optimized by lifestyle modification and metformin. Primary care physicians and endocrinologists widely prescribe SGLT2 inhibitors to manage persistent hyperglycemia. Empagliflozin can significantly reduce atherosclerotic cardiovascular mortality and morbidity in patients with T2DM. It is also prescribed for decreasing the progression of diabetic nephropathy, decreasing mortality in heart failure with reduced ejection fraction patients, and helping with weight loss [4]. Here we present a rare case of a 58-year-old obese diabetic female who presented necrotizing fasciitis using Sodium-Glucose Cotransporter-2 (SGLT2) inhibitor (SGLT-2), specifically Empagliflozin.

## Case presentation

A 58-year-old female with a past medical history of type 2 diabetes mellitus, hypertension, and hyperlipidemia presented to the emergency room with a complaint of severe pain, redness, and swelling in the right upper thigh and perineum for the past week. The patient was recently treated twice for a genital yeast infection one month ago. A review of the system was remarkable for a severely tender, indurated, erythematous, possible abscess-like lesion in the right upper thigh and pelvis, which was associated with fever and chills. Physical examination in the emergency department was significant for borderline low blood pressure of 106/53 mm/hg, tachycardia of 104 beats per minute, fever of 101 F, and BMI of 48.3 kg/m2. She looked toxic, with significantly tender, warm, erythematous, indurated crepitation on the perineum and area on the medial aspect of the right thigh with mild malodorous vaginal discharge.

The emergency department lab showed significant neutrophilic leukocytosis with a left shift of white blood cells (WBC) 26.6 x 10^3/ul, glucose 230 mg/dL. Chemistry showed low bicarbonate of 18 mmol/L, anion gap of 18, blood lactate was elevated 2.47 mmol/L, and a significantly elevated BUN of 35.7 mg/dL and creatinine (creatinine) Cr) 1.38 mg/dL, with low eGFR 44.4 mL/min. Arterial blood gas demonstrated normal venous pH of 7.40, and venous pCO_2_ was 35.4 mmHg. Patients' inflammatory markers were significantly elevated C-reactive protein (CRP) 27.0 mg/dL, erythrocyte sedimentation rate (ESR) 99 mm/hr and procalcitonin 2.01 ng/ml. Chemistry also showed deranged liver function tests (LFTs), aspartate aminotransferase (AST) 36 U/L, alanine aminotransferase (ALT) 83 U/L, and high alkaline phosphatase (ALK) 198.8 U/L. There was a decrease in total protein 5.6 g/dL, low albumin 2.7 g/dL, an increase in total bilirubin 2.5 mg/dL, and HbA1c 7.3 (Table 1).

**Table 1:** Summarized table for the laboratory values on admission :

Labs	Value	Reference
Glucose	230 mg/dL	70-105 mg/dL
Blood urea creatinine (BUN)	35.7 mg/dL	8.4-25.7 mg/dL
Creatinine	1.38 mg/dL	0.72-1.25 mg/dL
Sodium	134 mmol/L	136-145 mmol/L
Potassium	3.3 mmol/L	3.5-5.1 mmol/L
Bicarbonate	18 mmol/L	22.0-29.0 mmol/L
Albumin	2.8 g/dL	3.5-5.2 mg/dL
Bilirubin, total	2.4 mg/dL	0.2-1.2 mg/dL
pH venous	7.40	7.350-7.450 unit
Anion Gap	18	8-16
Serum lactate	2.47 mmol/L	0.50-1.90 mmol/L
Procalcitonin	2.01 ng/mL	0.00-0.08 ng/mL
C-Reactive protein	27.0	0.50-1.00 mg/dL
Alanine Aminotransferase (ALT)	77 U/L	10-55 U/L
Aspartate Aminotransferase (AST)	41 U/L	5-34 U/L
Alkaline Phosphatase (ALK)	198.8 U/L	40-150 U/L
Glomerular filtration rare (eGFR)	44.4 ml/min	>90 ml/min
White blood count	26.6 uL	4.5-11.0 uL
Neutrophils	90.2	40-70%
Hemoglobin A1c	7.3	4.8-5.6
Prothrombin time	17.5 sec	9.8-13.4 sec
Partial thromboplastin time	42.0 Sec	24.9-35.9 sec
International normalized ratio (INR)	1.43	0.85-1.15

The patient's urinalysis showed a Ph of 5.5, significant glucosuria >1000, mild ketonuria, and bilirubin (Table 2). Preliminary urine and two blood cultures grew gram-positive cocci in the chain. Emergency computed tomography (CT) pelvis and right lower extremity with contrast revealed infiltration of punctate bubbles of gas in the subcutaneous tissue in the pelvis, which extended to the medical right thigh and edema without discrete focal fluid collection consistent with necrotizing fasciitis or gangrenous infection of the perineum and right thigh (figure 10). The Sepsis protocol was activated, and the patient was started on intravenous fluid according to sepsis protocol (30 ml/kg) and broad-spectrum antibiotics, including vancomycin, meropenem, and clindamycin. She underwent emergency exploration by surgery with washout and application of a vacuum dressing in the right thigh. The post-operative diagnosis was FG and necrotizing fasciitis of the right thigh, which extended to the lower abdominal wall. The wound culture later grew moderate streptococcus viridans and moderate corynebacterium species. She had two more episodes of surgical debridement afterward. Post-procedure, the patient had improvement in WBC count. Total WBC count downtrend from 11.3 in 3 days. Her home medication, Empagliflozin, was immediately discontinued on admission, and she was started on a basal-bolus insulin regimen in the hospital for blood sugar optimization.

**Table 2:** Urine Analysis on admission:

Urinalysis	Value	Reference
Color	Dark yellow	clear
Glucose	>1000 mg/dL	Negative
ketones	Trace	Negative
Bilirubin	Moderate	Negative
Nitrate and Leukocyte esterase	Negative	Negative
White blood cell	2	0-5/ High power field

**Figure 1:**
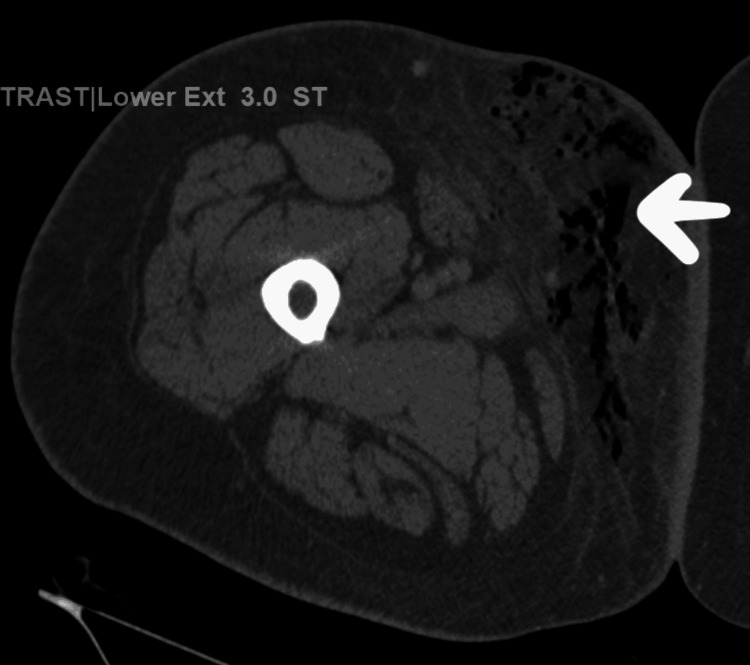
Computed tomography (CT) of the right lower extremity revealed punctate bubbles of gas in the subcutaneous tissue in the pelvis which extended to the medial right thigh consistent with NF ( arrow-marked area on the image)

## Discussion

There is an increase in the incidence of type 2 diabetes in the United States due to the obesity epidemic, and most patients require multiple drugs to control blood glucose adequately. The SGLT1 proteins are found in the small intestine, and SGLT2 proteins are found in the proximal convoluted tubule of the kidney. The botanical extract, phlorizin, has been identified as a non-specific inhibitor of Sodium-glucose cotransporter (SGLT) proteins by studying glucosuria in diabetic patients, subsequently confirming the inhibition of SGLT2 proteins is favorable to carbohydrate metabolism. SGLT2 inhibitors work by a novel mechanism that acts independently of insulin production. It reduces renal tubular glucose reabsorption by 90% without stimulating the pancreas to release insulin and thus lowers the blood glucose level [4]. SGLT2 inhibitors, specifically empagliflozin, which our patient took, have been widely prescribed due to their cardioprotective effects. The popularity of empagliflozin is due to its various benefits, such as lowering the rate of hospitalization in patients with both heart failure with reduced and preserved ejection fractions [5], reducing cardiovascular mortality and worsening heart failure with or without diabetes, decreased hemoglobin A1C levels, decreased weight, weight circumference, and decrease in blood pressure without reflex tachycardia as well as a decrease in serum uric acid. However, there are some limitations to using SGLT-2 inhibitors, which include frequent urinary tract infections by bacteria or genitourinary infections by yeast due to increased glycosuria. It can lower bone mineral density, resulting in a high bone fracture risk. However, one scarce and deadly adverse effect of SGLT2 inhibitors is FG. From 2013 to 2018, the FDA Adverse Events Reporting System (FAERS) database reported that there were 12 FG cases identified that were associated with SGLT2 inhibitor usage [6]. The cases were more common in males than females. American Diabetic Association, in January 2022, 491 cases of FG associated with SGLT2 inhibitor were identified. Out of 491 cases, canagliflozin caused 162 cases, dapagliflozin caused 101 cases, empagliflozin caused 223 cases, and ertugliflozin caused only 5 cases. While few studies showed no significant risk of necrotizing fascitis and FG with SGLT-2 inhibitors [7], it is concerning that the number of FG cases continued to rise with SGLT-2 inhibitor use despite the issuance of an FDA warning back in 2018 [8]. Therefore we think that it is paramount that the physician and the patient be on the lookout for early signs and symptoms of this devastating complication. 

The necrotizing fasciitis-the underlying mechanism of FG, also known as a flesh-eating bacterial infection, is a rapid-onset skin infection. In our case, the patient presented with a severe infection of the lower abdomen, right medial thigh, and perineum with significant leukocytosis, high fever, and hypotension. She rapidly decompensated in the emergency room and subsequently was taken into surgery for surgical exploration and debridement for Fournier gangrene with necrotizing fasciitis. Without emergent surgery and appropriate antibiotics, this can quickly progress to sepsis, septic shock, and even death. The etiology of necrotizing fasciitis in the patient above is a cutaneous breakdown in the perianal or genital region due to a recent yeast infection prior to hospitalization. SGLT2 inhibitors have become popular among physicians due to their well-known benefits for cardiovascular and renal protection. It is also essential to be aware of life-threatening complications due to the same sodium-proton antiporter proteins. There is no way to mitigate the adverse effects of SGLT2 inhibitors without reducing the benefits [9].

## Conclusions

Although there are many benefits of SGLT-2 inhibitors, the life-threatening complication, i.e., Fournier's gangrene, can be a rare side effect of SGLT-2 inhibitors, as seen in the above patient. It is of utmost importance to stop taking SGLT2 inhibitors if there is a concern of urinary tract infection or urogenital infection with yeast. Proper education and close follow-up with primary care or an endocrinologist are therefore critical while on SGLT-2 inhibitors. Overall, the benefits of SGLT-2 inhibitors outweigh the risks by many folds. However, physicians must be vigilant and focused on the safe use of SGLT2 inhibitors, especially before and after prescribing. Close follow-up and proper education about known side effects and future adverse effects that can develop because of comorbidity are required. Patients should be counseled to seek medical attention promptly if they experience any tenderness, erythema, or swelling in the perineal area or lower extremity while on this medication.
